# Relation Between Genotype and Behavioral Phenotype in Children and Adolescents With Neurofibromatosis Type 1

**DOI:** 10.1016/j.jaacop.2026.06.008

**Published:** 2026-07-01

**Authors:** Myrthe J. Ottenhoff, Andre B. Rietman, Daniela Gawehns, Mark Nellist, Shruti Garg, Julieta O’Flaherty, Susan J. Kimber, Emma M.M. Burkitt Wright, Caroline Wouters, Ellen Denayer, Rianne Oostenbrink, Pieter F.A. de Nijs, Leontine W. ten Hoopen, Marie-Claire Y. de Wit, Rick van Minkelen, Henriette A. Moll, Ype Elgersma, Gwendolyn C. Dieleman, Sabine E. Mous

**Affiliations:** a Erasmus MC Center of Expertise for Neurodevelopmental Disorders (ENCORE), Erasmus University Medical Center, Rotterdam, the Netherlands; b Erasmus Medical Center, Rotterdam, the Netherlands; c Erasmus Medical Center Sophia Children’s Hospital, Rotterdam, the Netherlands; d University of Manchester, Manchester, United Kingdom; e Royal Manchester Children's Hospital, Central Manchester University Hospitals NHS Foundation Trust, Manchester Academic Health Sciences Centre, Manchester, United Kingdom; f Full Member of the European Reference Network on Genetic Tumour Risk Syndromes (ERN GENTURIS), Nijmegen, the Netherlands; g Manchester University NHS Foundation Trust, Manchester, United Kingdom; h University Hospital Leuven, Leuven, Belgium; i Full member of the European Reference Network on Rare Congenital Malformations and Rare Intellectual Disability (ERN-ITHACA), Paris, France

**Keywords:** neurofibromatosis type 1, autism, attention-deficit/hyperactivity disorder, genotype–phenotype correlation

## Abstract

**Objective:**

Children with the autosomal dominant disorder neurofibromatosis 1 (NF1) exhibit a high prevalence of both attention-deficit/hyperactivity disorder (ADHD) (up to 40%) and autism (up to 11%). The genetic mechanisms underlying these behavioral phenotypes are largely unexplored. This study examines the associations between various *NF1* genotypes and NF1 behavioral phenotypes.

**Method:**

A retrospective cohort study was carried out in children and adolescents with NF1 from 3 national referral centers for NF1 (the Netherlands, United Kingdom, and Belgium). Anonymized data were collected on 5 primary outcomes to characterize the behavioral phenotype in NF1: autistic traits (Social Responsiveness Scale 2 Total T-score and Autism Diagnostic Observation Schedule Calibrated Severity Score), ADHD characteristics (Conners Inattention and Hyperactivity T-score), and global behavioral/emotional problems (Child Behavior Checklist Total Problems T-score). Genotypes were categorized based on 3 types of *NF1* variant definitions: coding variant type, variant effect (truncating vs nontruncating variants), and location within the gene.

**Results:**

In total, 408 children and adolescents with NF1 were included, of which 187 (46%) were girls. Range of median age at assessment across all outcomes was 6.0 to 8.4 years. Behavioral outcomes were not associated with any of the *NF1* genotype categories examined.

**Conclusion:**

In this large cohort, autism traits, ADHD characteristics, or global behavioral/emotional problems were not associated with *NF1* variant type, effect, or location. These findings indicate that the *NF1* variant classes examined here do not differentially influence the NF1 behavioral phenotype, although genotype–phenotype associations at the single-codon level cannot be ruled out.

## Plain language summary

Children with the genetic disorder neurofibromatosis 1 (NF1) may show characteristics of autism or attention-deficit/hyperactivity disorder (ADHD). This study looked at whether different NF1 gene mutations relate to the severity of these clinical characteristics. The study findings showed that no specific mutation was related to the number of autism or ADHD characteristics, suggesting that these mutations may have a similar effect on behavior. These findings highlight a need for more research to better understand how NF1 mutations relate to behavioral characteristics.

Children with the monogenic disorder neurofibromatosis 1 (NF1, MIM 162200) show an approximately 10-fold higher prevalence of both attention-deficit/hyperactivity disorder (ADHD) and autism spectrum disorder, hereafter autism^1^) relative to the general population: ADHD is diagnosed in up to 40% of NF1 patients^2^, ^3^, ^4^ and autism in up to 11%,^5^ with about 40% showing (sub)clinical autistic traits.^6^ Although the genetic mechanisms underlying the NF1 behavioral phenotype are largely unexplored, autistic traits in NF1 have been shown to strongly correlate within first-degree family pairs,^6^^,^^7^ suggesting that specific NF1 variants may differentially shape the behavioral phenotype (ie, genotype–phenotype associations).

NF1 is an autosomal dominant disorder with a birth incidence of ∼1:2,000,^8^ which is caused by heterozygous loss-of-function variants in *NF1* (MIM 613113). *NF1* encodes neurofibromin, a tumor suppressor protein that negatively regulates RAS signaling.^9^ Hallmark characteristics of NF1 are café-au-lait spots, (plexiform) neurofibromas, Lisch nodules, skinfold freckling, optic gliomas, and bone dysplasia. Additionally, individuals with NF1 are more likely to exhibit both challenges in global cognitive function^10^ and more specific cognitive challenges in areas such as visual perception and working memory^11^, ^12^, ^13^, ^14^, ^15^ as well as motor function.^15^

The severity of the somatic NF1 phenotype is unpredictable. Affected family members show a high variability of somatic characteristics.^16^^,^^17^ This variability can be understood from the important role for biallelic inactivation of *NF1* through stochastic second hit variants in the unaffected allele in affected tissues.^18^ Despite the important role of this noninherited, stochastic factor, several NF1 genotype–phenotype correlations have been identified.^19^, ^20^, ^21^, ^22^, ^23^, ^24^ In contrast, the cognitive NF1 phenotype shows a variability that is similar to the general population, without specific intragenic genotypes standing out.^10^ This suggests that cognitive function in NF1 might be uniformly affected by heterozygous *NF1* loss-of-function in addition to genetic background variability.

Previous studies found a high correlation of autistic traits within first-degree family pairs carrying the same *NF1* variant.^6^^,^^7^ Additionally, 1 study found that the proximity of a pathogenic *NF1* variant to the 3′ end of the gene was associated with increased autistic traits.^25^ These findings suggest the possibility of genotype–phenotype correlations where specific *NF1* pathogenic variants differentially influence the behavioral phenotype. However, studies to identify genetic determinants for the behavioral NF1 phenotype have been limited, most likely because integration of molecular genetic confirmation into routine clinical care is a recent development and because the availability of detailed behavioral data has been limited.

Specific genotype characterizations, such as variant type and location along the gene, are particularly relevant because different types of variants may impact protein function in distinct ways. For example, variants that are nontruncating (ie, compatible with protein formation) might retain partial or altered functionality compared with truncating variants for which protein production is typically absent or severely disrupted, making domain-specific effects less likely. Consequently, nontruncating variants are more likely to exhibit functional heterogeneity related to variant, particularly in relation to key functional domains.

In several monogenic conditions, such variant class characterizations have been found to impact behavioral or neurodevelopmental phenotypes. For example, loss-of-function *SCN2A* variants are associated with an autism-predominant phenotype, whereas gain-of-function variants are associated with an epilepsy-predominant presentation.^26^ Additionally, specific *SATB1* missense variants are linked to more severe intellectual disability than truncating variants.^27^ Even in population cohorts, carriers of rare variants in developmental disorder genes show measurable neurodevelopmental differences, with protein-truncating variants generally having stronger effects than missense variants.^28^

Together, these findings suggest that the behavioral phenotype in NF1 may also vary depending on specific variant characteristics. We hypothesize that nontruncating *NF1* variants are associated with a milder behavioral phenotype and that this effect may depend on their location within the gene relative to key functional domains. Therefore, the aim of the current study is to investigate whether specific genotype characteristics, including variant type, function, and location within the gene, are associated with the behavioral phenotype in individuals with NF1.

To address these questions, we performed a detailed characterization of the behavioral phenotype in a large, international, multicenter cohort of children and adolescents with a genetically confirmed diagnosis of NF1. Behavioral outcomes included measures of autism and ADHD characteristics as well as quantitative proxy ratings for global behavioral and emotional problems. We investigated the relationship of these measures with genotypes using 3 different genetic variant classifications: coding variant type, variant effect (truncating vs nontruncating variants), and location of the variant within the gene.

## Method

### Study Population

We performed retrospective data collection at 3 national NF1 outpatient clinics: (1) Erasmus MC Center of Expertise for Neurodevelopmental Disorders (ENCORE) at the Erasmus MC-Sophia Children’s Hospital, Rotterdam, the Netherlands; (2) the Manchester Centre for Genomic Medicine (MCGM) at the University of Manchester, Manchester, United Kingdom; and (3) the Center of Human Genetics (CHG) at the University Hospital Leuven, Leuven, Belgium. This study was approved by the institutional review board of each participating center (Medical Ethics Committee of the Erasmus Medical Center, Rotterdam; Greater Manchester Research Ethics Committee; and Ethics Committee Research UZ/KU Leuven). Written informed consent was formally waived as there was no patient burden and no privacy concern.

The periods of data collection aligned across centers and are specified in Table 1. We included individuals aged < 18 years with a clinical NF1 diagnosis based on the National Institutes of Health^29^ criteria and confirmed using genetic testing. Exclusion criteria were genetic testing indicating a variant of uncertain significance, segmental NF1, a genetically confirmed diagnosis of Legius syndrome, or a comorbid genetic disorder affecting neurocognitive development.

**Table 1:** Cohort Characteristics

	Erasmus MC Center of Expertise for Neurodevelopmental Disorders	Manchester Centre for Genomic Medicine	Center of Human Genetics	Total	Main effect of center		
	(n = 185)	(n = 105)	(n = 118)	(n = 408)	Statistic	*p*	Adjusted *p*
Data collection period	2011-2018	2010-2019	2006-2022				
Sex, female	86 (46.5)	46 (43.8)	55 (46.6)	187 (45.8)	χ^2^(2) = 0.2337	.8897	1
Inheritance					χ^2^(2) = 16.5771	.0003	.0025
De novo	125 (67.6)	50 (47.6)	73 (61.9)	248 (60.8)			
Familial	50 (27.0)	55 (52.4)	37 (31.4)	142 (34.8)			
Missing	10 (5.4)	0 (0)	8 (6.8)	18 (4.4)			
Variant type					χ^2^(10) = 22.661	.0121	.0845
Missense	29 (15.7)	11 (10.5)	20 (16.9)	60 (14.7)			
Frameshift	51 (27.6)	22 (21.0)	34 (28.8)	107 (26.2)			
Splicing	29 (15.7)	29 (27.6)	24 (20.3)	82 (20.1)			
Microdeletion	12 (6.5)	0 (0)	7 (5.9)	19 (4.7)			
Nonsense	53 (28.6)	29 (27.6)	21 (17.8)	103 (25.2)			
Other	11 (5.9)	14 (13.3)	12 (10.2)	37 (9.1)			
Variant expression effect^a^					χ^2^(2) = 1.4467	.4851	1
Truncating	104 (56.2)	51 (48.6)	69 (58.5)	224 (54.9)			
Nontruncating	33 (17.8)	11 (10.5)	24 (20.3)	68 (16.7)			
Not applicable	48 (25.9)	40 (38.1)	25 (21.2)	113 (27.7)			
Missing	0 (0)	3 (2.9)	0 (0)	3 (0.7)			
Variant GAP location^a^					χ^2^(4) = 1.7823	.7757	1
Pre-GAP	14 (7.6)	3 (2.9)	10 (8.5)	27 (6.6)			
Intra-GAP	9 (4.9)	5 (4.8)	6 (5.1)	20 (4.9)			
Post-GAP	10 (5.4)	3 (2.9)	8 (6.8)	21 (5.1)			
Not applicable	152 (82.2)	94 (89.5)	94 (79.7)	340 (83.3)			
Intracranial pathology					χ^2^(10) = 20.4394	.0254	.1667
None	144 (77.8)	97 (92.4)	93 (78.8)	334 (81.9)			
OPG asymptomatic	7 (3.8)	5 (4.8)	7 (5.9)	19 (4.7)			
OPG symptomatic	12 (6.5)	1 (1.0)	9 (7.6)	22 (5.4)			
Glioma asymptomatic	7 (3.8)	1 (1.0)	0 (0)	8 (2.0)			
Glioma symptomatic	6 (3.2)	0 (0)	3 (2.5)	9 (2.2)			
Other	9 (4.9)	1 (1.0)	6 (5.1)	16 (3.9)			
Psychoactive medication use					χ^2^(4) = 11.2575	.0238	.1429
None	157 (84.9)	90 (85.7)	99 (83.9)	346 (84.8)			
Attention-deficit/hyperactivity disorder	17 (9.2)	5 (4.8)	13 (11.0)	35 (8.6)			
Other	6 (3.2)	10 (9.5)	2 (1.7)	18 (4.4)			
Missing	5 (2.7)	0 (0)	4 (3.4)	9 (2.2)			
Social Responsiveness Scale, second edition							
Age,^b^ y	6.64 (5.45-8.73)	8.70 (7.10-11.3)	9.00 (6.00-12.0)	8.00 (6.00-11.0)	*F*(2,329) = 14.6437	<.0001	<.0001
Total T-score^c^	52.0 (46.0-62.0)	74.0 (62.0-85.0)	61.0 (52.0-72.0)	60.5 (50.3-75.0)	*F*(2,327) = 37.4785	<.0001	<.0001
Missing	49 (26.5)	0 (0)	29 (24.6)	78 (19.1)			
Autism Diagnostic Observation Scale							
Age,^b^ y	6.31 (4.56-8.34)	8.55 (7.85-10.1)	6.00 (5.00-10.0)	6.86 (5.19-9.29)	*F(*2,259) = 12.1258	<.0001	.0001
Module					χ^2^(6) = 23.0663	.0008	.007
Preverbal module	0 (0)	0 (0)	0 (0)	0 (0)			
Module 1	15 (8.1)	1 (1.0)	3 (2.5)	19 (4.7)			
Module 2	43 (23.2)	5 (4.8)	14 (11.9)	62 (15.2)			
Module 3	103 (55.7)	54 (51.4)	18 (15.3)	175 (42.9)			
Module 4	5 (2.7)	0 (0)	0 (0)	5 (1.2)			
Calibrated severity score^d^	2.00 (1.00-3.00)	6.00 (2.00-7.00)	4.00 (2.00-7.00)	2.00 (1.00-6.00)	*F*(2,248) = 24.6089	<.0001	<.0001
Missing	21 (11.4)	45 (42.9)	91 (77.1)	157 (38.5)			
Conners Rating Scale							
Age,^b^ y	6.64 (5.57-8.59)	8.70 (7.10-11.3)	NA	7.54 (5.95-9.81)	*F*(1,209) = 23.7196	<.0001	<.0001
Inattention T-score^d^	55.0 (48.0-66.5)	71.0 (60.0-79.5)	NA	61.0 (51.0-75.8)	*F*(1,212) = 36.9917	<.0001	<.0001
Hyperactivity T-score^d^	50.0 (44.0-60.0)	68.0 (56.0-82.5)	NA	56.0 (46.0-69.0)	*F*(1,214) = 60.1261	<.0001	<.0001
Missing	56 (30.3)	18 (17.1)	118 (100)	192 (47.1)			
Child Behavior Checklist							
Age,^d^ y	6.43 (5.27-8.65)	NA	6.00 (5.00-9.50)	6.40 (5.00-9.00)	*F*(1,251) = 0.0514	.8208	1
Total Problems T-score^d^	58.0 (47.5-66.5)	NA	59.0 (51.0-63.8)	58.0 (50.0-65.0)	*F*(1,247) = 0.012	.9128	1
Missing	30 (16.2)	105 (100)	24 (20.3)	159 (39.0)			

Note: Values are n (%) or median (interquartile range). GAP = GTPase activating protein domain; OPG = optic pathway glioma.

a Chi-square test does not include "not applicable" category.

b For analysis, this variable underwent logarithmic transformation.

c For analysis, this variable was first square-root transformed.

d For analysis, this variable was transformed into signed ranks.

Across all 3 centers, behavioral functioning was assessed using ≥1 of 4 standardized instruments: the Social Responsiveness Scale, second edition (SRS-2), the Conners Rating Scale (CRS), Autism Diagnostic Observation Schedule (ADOS), and Child Behavior Checklist (CBCL; described in detail below in Behavioral Outcomes). At ENCORE, all children were referred for a behavioral assessment as part of standard follow-up, regardless of any presumption of behavioral problems, and all behavioral measures included in this study were routinely administered. Because the assessments were embedded in routine clinical care rather than a research protocol, individual measures were occasionally missing for practical reasons. Data collected at ENCORE are part of an ongoing cohort study of children with NF1 in outpatient follow-up, and portions of these data have been reported in previous publications.^5^^,^^10^^,^^30^, ^31^, ^32^ At the MCGM, data were collected as part of 2 previously published clinical studies^33^^,^^34^ in which the SRS-2, CRS, and ADOS were administered to all participants; the CBCL was not part of either protocol. At the CHG, data were collected from children referred to the psychological services if psychological assessment was warranted based on parental reports. The specific measures administered to each child were selected by the clinician on the basis of the presenting concerns, which could include the ADOS, SRS-2, and CBCL but never included the CRS. A portion of the data collected at the CHG was also included in several previous publications.^32^^,^^35^^,^^36^ In all 3 centers, these data were supplemented with data of children that visited each center solely for the purpose of participating in a research study.

Because recruitment procedures and assessment protocols differed across centers, some degree of intercenter heterogeneity in cohort characteristics and behavioral symptom severity is expected. However, given the rarity of NF1, a multicenter approach was necessary to achieve an adequate sample size.

### Behavioral Outcomes

Five primary outcome measures were chosen to reflect 3 domains: autism characteristics, ADHD characteristics, and global behavioral and emotional problems. Autism characteristics were assessed with 2 different instruments: the SRS-2, a parent-rated questionnaire assessing presence and severity of behaviors associated with autism,^37^ and the ADOS, a semistructured observational instrument.^38^, ^39^, ^40^ For the ADOS, both the ADOS-G and ADOS-2 were used, of which the ADOS-2 was applied in most cases (78%). All ADOS-G assessments were scored according to the updated ADOS-2 criteria. The ADOS was administered by a trained researcher or certified psychologist. The specific primary outcomes for autism characteristics are defined as the SRS-2 Total T-score and the ADOS Calibrated Severity Score (ADOS CSS).

ADHD characteristics were assessed with the parent-rated version of the CRS, an ADHD screening questionnaire. Both the CRS Revised version^41^ and version 3^42^ were used, of which the Revised version was most common (88%). Because the CRS does not provide a total score and the subscales differ between the 2 versions, we defined 2 primary ADHD outcomes: CRS Inattention, based on either the Cognitive problems/Inattention subscale T-score (Revised) or the Inattention subscale T-score (version 3), and CRS Hyperactivity, based on the Hyperactivity subscale T-score (Revised) or the Hyperactivity/Impulsivity subscale (version 3).

For the assessment of a broad spectrum of behavioral and emotional problems, the parent-rated CBCL from the Achenbach System of Empirically Based Assessment was used.^43^^,^^44^ The CBCL primary outcome was defined as the Total Problems T-score. No specific secondary outcomes were predefined, but exploratory analyses of subscales were permitted if primary outcomes yielded significant results.

### *NF1* Genotype and Variant Classification

*NF1* variant screening and assignment of pathogenicity level were performed in a diagnostic setting using DNA-based molecular techniques as previously described.^45^ Pathogenic variants were subsequently classified according to their predicted coding variant type into 6 categories: missense, frameshift, splicing, nonsense, chromosomal microdeletions (CMDs; including types I-III and atypical deletions), or other variant types (comprising small deletions [involving <17 codons], large deletions [≥17 codons], insertions, and duplications). Methods for variant classification according to the above-described categories were as described previously.^10^

Additionally, all intragenic variants were classified as being likely truncating or not. We defined truncating as a variant that is predicted to be incompatible with protein production, creating 2 groups: truncating (ie, likely no protein production, *NF1*x group) or nontruncating (ie, likely protein production, *NF1*p group). Frameshift and nonsense variants (including nonsense variants in the last *NF1* exon [n = 2]) were classified as *NF1*x, in addition to large and/or out-of-frame insertions, deletions, and duplications. Missense variants and small in-frame insertions and deletions (indel; <17 codons) were classified as *NF1*p, unless an indel created a stop codon (classified as *NF1*x). We excluded splicing variants from this classification because their effect on protein expression is heterogeneous and cannot always be reliably predicted. Depending on the specific splicing effect, they may lead to exon skipping, frameshifts with premature termination, or in-frame alterations. Because these outcomes can correspond to either truncating or nontruncating effects, confident assignment to the *NF1*x or *NF1*p group was not possible. CMDs were excluded from this classification because these deletions also involve (partial) deletion of genes neighboring *NF1*.

Finally, we focused on the relation between the location of a variant within *NF1* and behavioral outcomes, motivated by a study by Morris and Gutmann^25^ showing higher SRS-2 scores in individuals with *NF1* variants located at the 3′ end of the gene. Although CMDs or splicing variants were excluded from the study, no distinction was made between variants being truncating or not. Because location-dependent functional effects are less biologically plausible for truncating variants that are expected to result in absent protein products, we visualized the relation between the variant location and behavioral outcome separately for the *NF1*x and *NF1*p groups.

As an additional variant location–based analysis, we classified variants in the *NF1*p group based on their location relative to the neurofibromin GTPase activating protein (GAP) domain using a similar approach to Morris and Gutmann,^25^ who compared behavioral scores of individuals with variants located before the GAP domain (5′ end) vs those after the GAP domain (3′ end). However, because the GAP domain plays a central role in the molecular mechanisms underlying NF1 symptoms,^46^ our analysis also included the variants in the GAP domain as a separate location category. We therefore defined 3 GAP location categories for this analysis: pre-GAP included variants affecting codons before codon 1198; intra-GAP referred to variants affecting codons 1198 to 1530; and post-GAP indicated variants affecting codons after codon 1530 (codon numbering based on NF1 transcript NM_000267.3 [RefSeq, NCBI]).

### Statistical Analysis

Although no formal a priori sample size calculation was performed, the study sample in the current study exceeds (for the 2-group analysis of variant effect [n = 128]) or closely approximates (for the 6-group analysis of coding variant [n = 216]) the total group size needed to detect a medium effect size (Cohen’s *d* = 0.5 or *f* = 0.25) with an 80% power using a 2-tailed test at *α* = 0.05, as estimated using G∗Power 3.1. However, group sizes did not meet the required total group size for the 3-group analysis of variant location within the *NF1*p subgroup (n = 159), whereas with the current sample sizes these analyses had a 23% to 36% power to detect a medium effect.

To estimate the effect of genotype on the 5 primary outcomes of behavioral function described above, we used random-effects models. Fixed effects were defined as either of the 3 different genotype classifications: variant type, variant effect (truncating or not), and variant location within the gene. Random effects were defined as intercepts per center (ENCORE, MCGM, or CHG) to account for outcome variability across centers.

We assessed the normality of residuals using Q-Q plots and histograms, exploring whether a square-root or base-10 logarithmic transformation improved the fit. Transformations that appeared visually acceptable were further evaluated for normality using the Kolmogorov-Smirnov test. If non-normality persisted despite transformations, we applied a nonparametric model based on ranked outcomes.

Descriptive variables were compared using χ^2^ tests for categorical data and linear regression for continuous variables. Linear models for descriptive data were evaluated for residual normality following the same procedure as the outcome analysis, and the same transformations or nonparametric methods were applied where necessary.

Within each of the 3 genotype analyses (variant type, truncating or not, and variant location), we adjusted *p*-values for multiple testing across the 5 primary outcomes. Adjustment for *p*-value was performed using the Holm-Bonferroni correction, with statistical significance defined as an adjusted *p* < .05. Because the primary outcomes are primarily total instrument scores, we applied a hierarchical strategy where exploratory analysis of subscales was allowed if the corresponding total score reached significance after correction across the 5 primary outcomes to limit type I error inflation. For analysis of descriptive variables, Holm-Bonferroni correction was performed separately for sets of *p*-values belonging to an individual descriptive table.

All analyses were performed using *R* programming language (version 4.2.3)^47^ in *RStudio* (version 2023.6.1.524)^48^ supplemented with the *lme4* package (version 1.1.32)^49^ to perform the random models and the *bestNormalize* package (version 1.9.0)^50^ to perform the outcome transformations.

## Results

### Cohort Characteristics

We included 408 children and adolescents across centers, of whom 45.8% were girls. The median age at assessment across the 5 primary behavioral outcomes ranged from 6.4 years to 8.0 years. For the SRS-2, ADOS, and CRS, age at assessment was unbalanced across cohorts (Table 1). Additionally, the NF1 inheritance pattern differed between cohorts, with the ENCORE and CHG cohorts having a majority of de novo NF1 cases (68% and 62%, respectively), in contrast to the MCGM cohort (48% de novo cases). The outcome scores for the SRS-2, ADOS, and CRS were significantly higher in the MCGM cohort compared with CHG (intermediate) and ENCORE (lowest). We did not find any differences concerning sex, variant type, variant expression effect, variant location within the gene, intracranial pathology, psychoactive medication use, CBCL age at assessment, or CBCL Total Problems T-score (Table 1). Across cohorts, the proportions of subclinical and clinical scores for each primary outcome were substantially higher than those expected in the general population (Table 2). Additionally, all 5 primary outcomes were moderately to strongly correlated (*r*_S_ = 0.30-0.73; all *p* < .001) (Figure S1, available online).

**Table 2:** Prevalence of Subclinical and Clinical Scores Among Individuals With Available Primary Outcome Data

	Subclinical^a^	Clinical
Social Responsiveness Scale, second edition, Total T-score		
Cutoff	≥60	≥76
No. (%)	169 (51.2)	82 (24.8)
Autism Diagnostic Observation Scale Calibrated Severity Score		
Cutoff	≥4	≥6
No. *(%*)	86 (34.3)	66 (26.3)
Conners Rating Scale Inattention T-score		
Cutoff	≥60	≥70
No. (%)	113 (52.8)	71 (33.2)
Conners Rating Scale Hyperactivity T-score		
Cutoff	≥60	≥70
No. (%)	94 (43.5)	52 (24.1)
Child Behavior Checklist Total Problems T-score		
Cutoff	≥60	≥64
No. (%)	116 (46.6)	71 (28.5)

Note:

a Meaning at least subclinical.

### Coding Variant Classification

Variant types, including missense, frameshift, splicing, CMD, nonsense, and other variants (small and large insertions, deletions, or duplications), were analyzed for potential differences in behavioral outcomes. We found no differences in population characteristics across these variant groups (Table S1, available online). None of the behavioral outcomes differed across variant type: SRS-2 Total T-score (*F*_(5, 322.01)_ = 1.32; *p* = .25; *p*_adjusted_ = 1.00), ADOS CSS (*F*_(5, 243.2)_ = 1.15; *p* = .34*; p*_adjusted_ = 1.00), CRS Inattention T-score (*F*_(5, 207.01)_ = 1.9; *p* = .10*; p*_adjusted_ = .48), CRS Hyperactivity T-score (*F*_(5, 209.01)_ = 1.25; *p* = .29; *p*_adjusted_ = 1.00), and CBCL Total Problems T-score (*F*_(5, 242.01)_ = 0.39; *p* = .86*; p*_adjusted_ = 1.00) (Figure 1 and Table 3).

**Figure 1:**
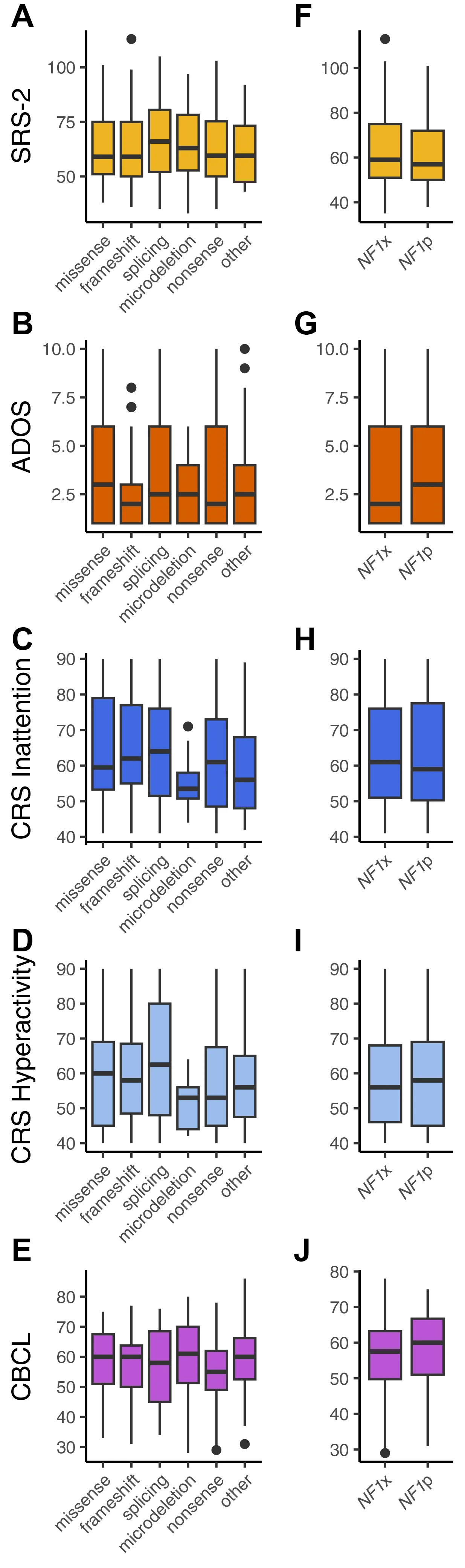
Boxplots of Primary Outcomes per 2 Different Genotype Classifications ***Note:** (A-E) Primary outcomes across variant type. (F-J) Primary outcomes by truncating (NF1x) or nontruncating variants (NF1p). ADOS = Autism Diagnostic Observation Scale Calibrated Severity Score; CBCL = Child Behavior Checklist Total Problems T-score; CRS Inattention = Conners Rating Scale Inattention T-score; CRS Hyperactivity = Conners Rating Scale Hyperactivity T-score; SRS-2 = Social Responsiveness Scale, second edition, Total T-score.*

**Table 3:** Primary Outcomes per Variant Type

Outcome	Estimate [95% CI]	No. of cases	Statistic	*p*	Adjusted *p*
Social Responsiveness Scale, second edition, Total T-score^a^		330	*F*_(5,322.01)_ = 1.32	.2548	1
Missense	8 [7.34-8.66]				
Frameshift	7.93 [7.61-8.25]				
Splicing	8.01 [7.66-8.35]				
Microdeletion	8.37 [7.82-8.92]				
Nonsense	7.85 [7.52-8.18]				
Other	7.68 [7.27-8.1]				
Autism Diagnostic Observation Scale Calibrated Severity Score^b^		251	*F*_(5,243.2)_ = 1.15	.3364	1
Missense	162.44 [117.43-207.45]				
Frameshift	130.91 [104.33-157.5]				
Splicing	143.21 [114.52-171.89]				
Microdeletion	146.24 [107.56-184.92]				
Nonsense	146.52 [120.03-173.02]				
Other	140.43 [103.54-177.32]				
Conners Rating Scale Inattention T-score		214	*F*_(5,207.01)_ = 1.9	.0952	.4762
Missense	67.46 [54.98-79.94]				
Frameshift	67.01 [61.22-72.81]				
Splicing	64.03 [57.85-70.21]				
Microdeletion	60.95 [50.6-71.3]				
Nonsense	62.49 [56.71-68.27]				
Other	58.19 [50.56-65.81]				
Conners Rating Scale Hyperactivity T-score^b^		216	F_(5,209.01)_ = 1.25	.2876	1
Missense	123.46 [60.39-186.52]				
Frameshift	122.67 [98.86-146.47]				
Splicing	119.73 [94.3-145.16]				
Microdeletion	110.13 [69.28-150.98]				
Nonsense	102.67 [78.92-126.41]				
Other	100.94 [69.46-132.41]				
Child Behavior Checklist Total Problems T-score		249	F_(5,242.01)_ = 0.39	.858	1
Missense	57.62 [54.06-61.18]				
Frameshift	56.59 [52.13-61.06]				
Splicing	56.5 [51.52-61.48]				
Microdeletion	58.79 [51.66-65.91]				
Nonsense	55.14 [50.44-59.84]				
Other	58.33 [51.83-64.84]				

Note: ^a^For analysis, this variable was square-root transformed.

b For analysis, this variable was transformed into signed ranks.

### Variant Effect Classification

We examined the relationship between genotype and behavioral outcomes by focusing on a variant being truncating (*NF1*x group, n = 224) or nontruncating (*NF1*p group, n = 68). These groups did not differ in terms of sex, age at assessment, or any other demographics (Table S2, available online). We found no difference in behavioral outcome scores between the *NF1*x and *NF1*p group: SRS-2 Total T-score (*F*_(1, 207.16)_ = 0.13; *p* = .72; *p*_adjusted_ = 1.00), ADOS CSS (*F*_(1, 178.07)_ = 0.07; *p* = .07; *p*_adjusted_ = .36), CRS Inattention T-score (*F*_(1, 146.22)_ = 0.17; *p* = .68; *p*_adjusted_ = 1.00), CRS Hyperactivity T-score (*F*_(1, 147.11)_ = 0.87; *p* = .35; *p*_adjusted_ = 1.00), and CBCL Total Problems T-score (*F*_(1, 197.28)_ = 0.42; *p* = .52; *p*_adjusted_ = 1.00) (Figure 1 and Table 4).

**Table 4:** Primary Outcomes by Truncating or Nontruncating Variant

Outcome	Estimate [95% CI]	No. of cases	Statistic	p	Adjusted *p*
Social Responsiveness Scale, second edition		211	*F*_(1,207.16)_ = 0.13	.7204	1
*NF1*x	63.86 [59.06-68.66]				
*NF1*p	64.74 [54.88-74.6]				
Autism Diagnostic Observation Scale Score^a^		182	*F*_(1,178.07)_ = 3.27	.0725	.3624
*NF1*x	132.75 [112.12-153.38]				
*NF1*p	151.77 [108.05-195.49]				
Conners Rating Scale Inattention^a^		149	*F*_(1,146.22)_ = 0.17	.6815	1
*NF1*x	108 [86.54-129.47]				
*NF1*p	112.51 [65.16-159.87]				
Conners Rating Scale Hyperactivity^a^		150	*F*_(1,147.11)_ = 0.87	.3531	1
*NF1*x	106.75 [87.42-126.08]				
*NF1*p	115.94 [53.7-178.19]				
Child Behavior Checklist^a^		200	F_(1,197.28)_ = 0.42	.5173	1
*NF1*x	117.49 [96.22-138.75]				
*NF1*p	124.54 [106.31-142.77]				

Note: NF1p, nontruncating; NF1x, truncating.

a For analysis, this variable was transformed into signed ranks.

### In-Gene Variant Location

Visualization of the relation between the variant location and behavioral outcome separately for the *NF1*x and *NF1*p groups did not suggest a location-based effect for the *NF1*x group (Figure 2). However, for the *NF1*p group, we observed an increase for some behavioral outcomes, particularly the CRS Inattention and Hyperactivity T-scores, for variants towards the 3′ end of *NF1*.

**Figure 2:**
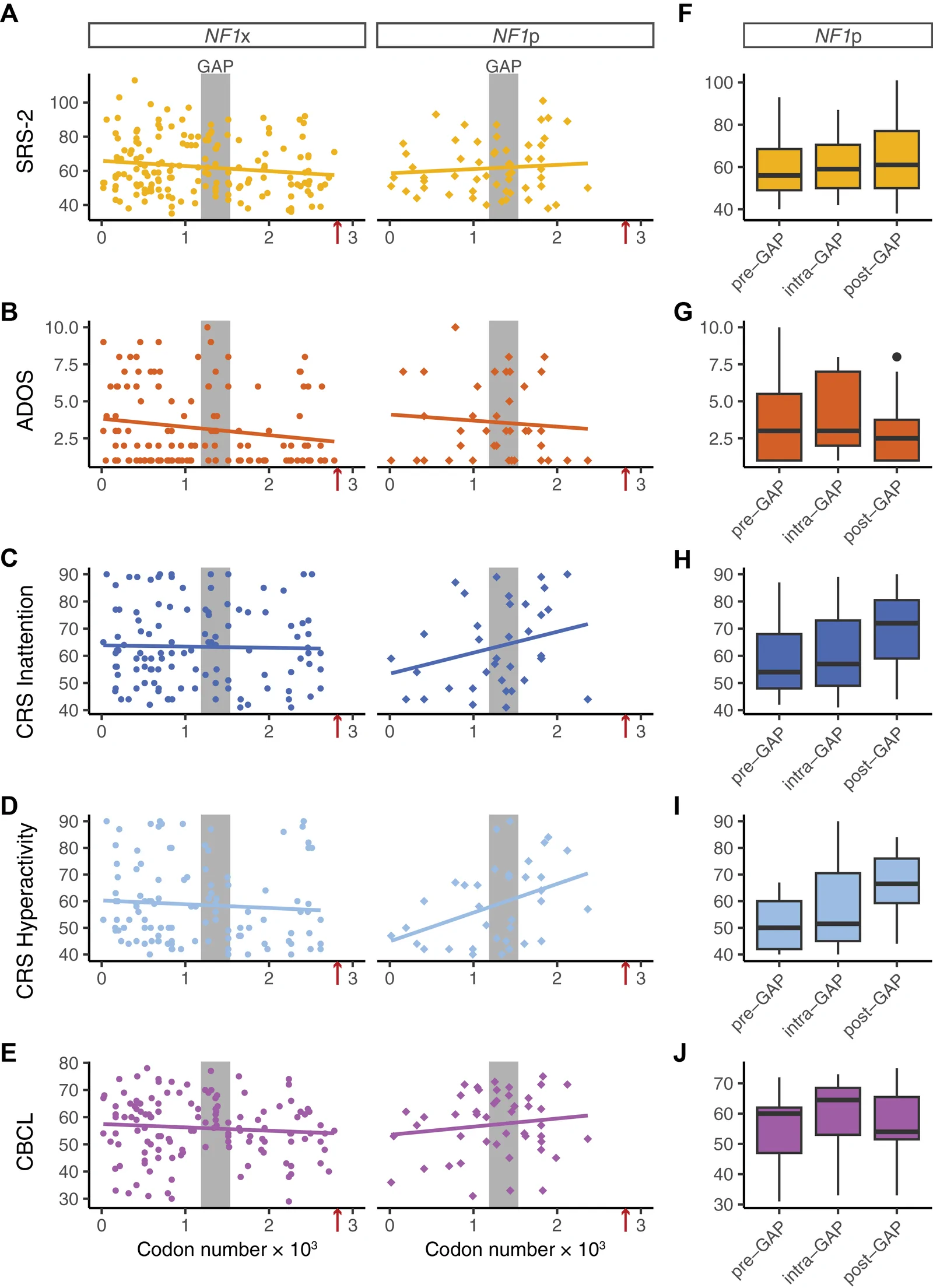
Primary Outcomes by Variant Location ***Note:****(A-E) Scatterplots showing primary outcomes by variant codon location within the gene, separately for truncating (NF1x) and nontruncating variants (NF1p). Gray areas are the GAP domain. Red arrows on the x-axis indicate the C-terminal amino acid (corresponding to codon 2818). (F-J) Boxplots of primary outcomes in the NF1p group per variant location category, relative to the GAP domain: pre-, intra-, or post-GAP domain location. ADOS = Autism Diagnostic Observation Scale Calibrated Severity Score; CBCL = Child Behavior Checklist Total Problems T-score; CRS Inattention = Conners Rating Scale Inattention T-score; CRS Hyperactivity = Conners Rating Scale Hyperactivity T-score; GAP = GTPase activating protein domain; SRS-2 = Social Responsiveness Scale, second edition, Total T-score.*

Additionally, we compared outcomes between pre-GAP, intra-GAP, and post-GAP locations. This analysis was performed for the *NF1*p group only, as only nontruncating variants are expected to retain residual functionality (as discussed in the introduction and Method). No significant differences in sex, age at assessment, or other demographic variables were observed across these location groups (Table S3, available online). Analyzing behavioral outcomes across GAP location groups revealed no significant differences: SRS-2 Total T-score (*F*_(2, 53.52)_ = 0.11; *p* = .90; *p*_adjusted_ = 1.00), ADOS CSS (*F*_(2, 35.94)_ = 0.30; *p* = .74; *p*_adjusted_ = 1.00), CRS Inattention T-score (*F*_(2, 22.92)_ = 1.86; *p* = .18; *p*_adjusted_ = .72), CRS Hyperactivity T-score (*F*_(2, 26.63)_ = 2.91; *p* = .07; *p*_adjusted_ = .36), and CBCL total T-score (*F*_(2, 42.08)_ = 0.77; *p* = .47; *p*_adjusted_ = 1.00) (Figure 2 and Table 5).

**Table 5:** Primary Outcomes by Variant Location for the *NF1*p Group

Outcome	Estimate [95% CI]	No. of cases	Statistic	*p*	Adjusted *p*
Social Responsiveness Scale, second edition, Total T-score^a^		57	*F*_(2,53.52)_ = 0.11	.8958	1
Pre-GAP	27.92 [20.88-34.97]				
Intra-GAP	29.01 [18.04-39.97]				
Post-GAP	30.43 [20.19-40.67]				
Autism Diagnostic Observation Scale Calibrated Severity Score^a^		39	*F*_(2,35.94)_ = 0.3	.7439	1
Pre-GAP	19.79 [13.83-25.75]				
Intra-GAP	21.81 [13.22-30.4]				
Post-GAP	18.29 [9.52-27.06]				
Conners Rating Scale Inattention T-score^a^		36	*F*_(2,22.92)_ = 1.86	.1788	.7153
Pre-GAP	15.58 [10-21.16]				
Intra-GAP	16.91 [8.66-25.15]				
Post-GAP	23.12 [15.07-31.18]				
Conners Rating Scale Hyperactivity T-score^a^		37	*F*_(2,26.63)_ = 2.91	.0718	.359
Pre-GAP	13.88 [8.34-19.43]				
Intra-GAP	19.29 [11.29-27.29]				
Post-GAP	24.25 [16.25-32.25]				
Child Behavior Checklist Total Problems T-score^a^		46	*F*_(2,42.08)_ = 0.77	.4685	1
Pre-GAP	21.18 [15.12-27.24]				
Intra-GAP	27.38 [17.63-37.12]				
Post-GAP	23.33 [14.21-32.46]				

Note: GAP = GTPase activating protein domain.

a For analysis, this variable was transformed into signed ranks.

## Discussion

In this study, we examined the relationship between *NF1* genotype and behavioral phenotype (specifically autism characteristics, ADHD characteristics, and broader behavioral and emotional characteristics) in a cohort of 408 children and adolescents with NF1, representing the largest genotype–phenotype study focusing on behavior in NF1 to date. We found no significant associations between behavioral outcomes and the 3 *NF1* genotype classifications we investigated: variant type, truncating (*NF1*x) vs nontruncating variants (*NF1*p), and variant location within *NF1*.

The absence of an association between *NF1* variant type and behavioral outcomes contrasts the behavioral NF1 phenotype with the cognitive phenotype, which tends to be more severe in individuals with an *NF1* CMD.^20^^,^^51^^,^^52^ Combined with previous evidence that the behavioral and cognitive NF1 phenotype are only partially correlated,^53^ these findings are in line with findings in the general population showing a much lower genetic heritability of behavioral outcomes vs cognitive outcomes.^54^

The behavioral outcomes examined here showed a moderate to strong within-individual correlation between ADHD and autism characteristics, partly consistent with a prior report of the SRS-2 scores correlation of 0.61 with ADHD characteristics in children with NF1.^6^ This suggests that the autism trait burden largely coincides with the ADHD trait burden in children with NF1. However, the observational ADOS measures correlated only moderately with ADHD measures (0.31-0.43) relative to the SRS-2, which could indicate that stronger correlations between parent-rated questionnaires may partly reflect shared method variance next to common etiological underpinnings.

We hypothesized that individuals with nontruncating *NF1* variants (*NF1*p) might exhibit a milder behavioral phenotype, because neurofibromin produced from the variant allele could retain some residual function. Alternatively, we considered the possibility that the *NF1*p group might show increased severity or variability in behavioral scores if it included variants with additional negative effects beyond the loss-of-function effect, known as dominant negative variants.^55^ However, the lack of observed differences to individuals with truncating *NF1* variants (*NF1*x) suggests that most nontruncating *NF1* variants do not retain sufficient residual function to influence the behavioral phenotype and are therefore most likely pure loss-of-function variants. This interpretation is supported by structural analysis of neurofibromin, which reveals a complex conformation that explains why any pathogenic *NF1* variant is likely to result in a loss-of-function as opposed to a gain-of-function effect.^56^

In contrast to earlier research, which reports a positive correlation between a variant’s proximity to the 3′ end of *NF1* and autistic trait burden,^25^ we found no relation between the location of a pathogenic *NF1* variant and the severity of any of the behavioral outcomes. In this earlier study, a smaller sample size (n = 45) consisting primarily of individuals with truncating variants (93%) was assessed. In contrast, in the current study, we did not observe such location-dependent effects on behavioral outcomes in individuals with either truncating (n = 224) or nontruncating (n = 68) *NF1* variants.

The GAP domain contains the catalytic, active site of neurofibromin and is therefore responsible for the negative regulation of RAS signaling, a critical mechanism that defines *NF1* as a tumor suppressor gene. Apart from oncogenic consequences, disruption of this function has been linked to altered neuronal activity and behavioral deficits in NF1 mouse models.^57^, ^58^, ^59^, ^60^ We therefore wanted to investigate whether the behavioral phenotype in individuals with nontruncating variants located within the GAP domain would differ from those with variants outside the domain, although our results did not show such a GAP domain–specific association. These results reinforce the notion that most nontruncating *NF1* variants lack significant residual function, regardless of their specific location within the gene.

Both our findings on variants being truncating or not and on variant location are consistent with previous results regarding the cognitive NF1 phenotype, which also showed no significant differences across these genotype classifications.^10^ Interestingly, however, certain *NF1*p variants have been found to be related to a specific somatic phenotype. These associations primarily involve specific codons or short sequences of codons rather than broader regions of the gene: the *NF1* codons 844 to 848, p.Met992del, p.Met1149, p.Arg1276, p.Lys1423, and p.Arg1809.^19^^,^^21^, ^22^, ^23^, ^24^ Our cohort, unfortunately, did not contain enough individuals per genotype (median of 2.5 individuals [range 0-6] across the 6 mentioned genotypes) to investigate if this association also extends into the behavioral domain. Furthermore, because neuronal neurofibromin also has RAS-independent functions,^61^, ^62^, ^63^, ^64^, ^65^ it is possible that codon-specific genotypes relating to the behavioral phenotype do not overlap with the previously identified variants that differentially affect the somatic phenotypes.

However, to identify such new codon-specific phenotypic associations, much larger cohorts will be required (see the discussion in Ottenhoff *et al.*^10^) that include multiple individuals sharing the identical codon-specific variants. Such studies should distinguish between unrelated and family-related individuals as well as unaffected relatives to help clarify the contribution of genetic background vs the specific variant to the behavioral phenotype. These studies could significantly benefit our understanding of the underlying mechanisms of NF1-related behavioral problems.

A major strength of this study is its high subject number, which is, to our knowledge, the largest NF1 genotype–phenotype cohort including both detailed genetic and behavioral data to date and also exceeds the group size typically needed to detect medium effect sizes in most of the analyses conducted. Therefore, the absence of observed genotype–phenotype associations in this study is unlikely to be due to small group sizes.

A possible limitation to this study is the imbalances in severity of behavioral outcome measures and the age at assessment between centers, which could indicate selection bias arising from recruitment differences between centers. For example, at ENCORE, behavioral measures were assessed as part of standard outpatient care in all individuals, whereas at the MCGM and CHG, certain assessments were only conducted in response to parental concerns or within the context of clinical studies. However, if the severity of behavioral outcomes was strongly associated with certain genotype groups, we would expect to see differences in genotype proportions across centers. These were not observed for any of the 3 genotype classifications. Additionally, to address potential center-related differences, we included center as a random effect in our linear mixed models, reducing the potential confounding due to center effects.

An additional limitation is the uncertainty in our variant effect classification.^66^^,^^67^ To reduce potential misclassification, splicing variants were excluded from this analysis because of their uncertain effect on gene expression. Future studies might benefit from incorporating functional analyses of splicing variants, which could to help retain these variants for analyses and increase statistical power.

Finally, this study did not include analyses based on formal neurodevelopmental diagnoses. The dimensional behavioral measures used in this study capture variation across the full spectrum of autism- and ADHD-related traits and may therefore be more sensitive to subtle phenotypic differences than dichotomous diagnostic outcomes, especially because many children with NF1 show subclinical traits.^5^ However, elevated scores on these measures do not directly correspond to clinical diagnoses. Therefore, future studies could complement dimensional behavioral measures with systematically collected diagnostic information to clarify how genotype effects relate to clinical diagnoses. Including measures of adaptive and cognitive functioning would also be valuable in such studies, because these provide important information on the functional impact of behavioral traits and are commonly considered when evaluating neurodevelopmental conditions.

In conclusion, in this large NF1 genotype–phenotype cohort, we did not find any association with *NF1* variant classifications, including variant type, variant effect (truncating or nontruncating), and variant location within the gene, and behavioral outcomes in children and adolescents with NF1. These results differ from investigations on the cognitive phenotype in NF1, which is found to be more severe in individuals with a CMD.^10^ Our findings suggest that genetic influences on the NF1 behavioral phenotype do not occur at the level of broad *NF1* variant classifications but likely involve interactions at specific codons, as has been described for NF1 somatic phenotypes.

## CRediT authorship contribution statement

**Myrthe J. Ottenhoff:** Writing – review & editing, Writing – original draft, Visualization, Software, Project administration, Methodology, Investigation, Formal analysis, Data curation, Conceptualization. **Andre B. Rietman:** Writing – review & editing, Supervision, Methodology, Investigation, Conceptualization. **Daniela Gawehns:** Writing – review & editing, Investigation, Data curation. **Mark Nellist:** Writing – review & editing, Supervision, Resources, Methodology, Investigation. **Shruti Garg:** Writing – review & editing, Supervision, Project administration, Methodology, Investigation, Conceptualization. **Julieta O’Flaherty:** Writing – review & editing, Investigation, Data curation. **Susan J. Kimber:** Writing – review & editing, Supervision, Methodology, Investigation. **Emma M.M. Burkitt Wright:** Writing – review & editing, Supervision, Methodology, Investigation. **Caroline** **Wouters:** Writing – review & editing, Investigation, Data curation. **Ellen Denayer:** Writing – review & editing, Supervision, Project administration, Investigation, Conceptualization. **Rianne Oostenbrink:** Writing – review & editing, Methodology, Investigation. **Pieter F.A. de Nijs:** Writing – review & editing, Investigation. **Leontine W. ten Hoopen:** Writing – review & editing, Investigation. **Marie-Claire Y. de Wit:** Writing – review & editing, Supervision, Investigation. **Rick van Minkelen:** Writing – review & editing, Methodology, Investigation. **Henriette A. Moll:** Writing – review & editing, Supervision, Methodology. **Ype Elgersma:** Writing – review & editing, Supervision, Methodology, Funding acquisition. **Gwendolyn C. Dieleman:** Writing – review & editing, Supervision, Methodology, Investigation, Funding acquisition, Conceptualization. **Sabine E. Mous:** Writing – review & editing, Supervision, Project administration, Methodology, Investigation, Funding acquisition, Formal analysis, Conceptualization.
