# Supplementary material for: Relation Between Genotype and Behavioral Phenotype in Children and Adolescents With Neurofibromatosis Type 1
Source: JAACAP Open. 2026 Jul 1;4(5):768–82. doi: 10.1016/j.jaacop.2026.06.008 (PMC13643462; doi:10.1016/j.jaacop.2026.06.008)
Supplement: Supplementary file 1 [file mmc1.docx]

## Supplement

**Supplemental Figure 1**: Correlation between primary outcomes.

Correlation plot in which the circle size and colors corresponding to the Spearman correlation coefficient shown in each cell. All correlation coefficients had an unadjusted significance level of <0.001.

**Supplemental Table 1**: Cohort characteristics per variant type.

|  | **Missense** | **Frameshift** | **Splicing** | **microdeletion** | **nonsense** | **other** | **Comparisons** |  |  |
| --- | --- | --- | --- | --- | --- | --- | --- | --- | --- |
|  | **(*n* = 60)** | **(*n* = 107)** | **(*n* = 82)** | **(*n* = 19)** | **(*n* = 103)** | **(*n* = 37)** | **Statistic** | ***p*-value** | **Adjusted *p*-value** |
| **Sex** |  |  |  |  |  |  | *χ2*(5) = 6.0481 | 0.3016 | 1 |
| male | 31 (51.7%) | 49 (45.8%) | 46 (56.1%) | 10 (52.6%) | 64 (62.1%) | 21 (56.8%) |  |  |  |
| female | 29 (48.3%) | 58 (54.2%) | 36 (43.9%) | 9 (47.4%) | 39 (37.9%) | 16 (43.2%) |  |  |  |
| **Inheritance** |  |  |  |  |  |  | *χ2*(5) = 15.1067 | 0.0099 | 0.0892 |
| de novo | 44 (73.3%) | 64 (59.8%) | 42 (51.2%) | 16 (84.2%) | 66 (64.1%) | 16 (43.2%) |  |  |  |
| familial | 15 (25.0%) | 37 (34.6%) | 38 (46.3%) | 2 (10.5%) | 34 (33.0%) | 16 (43.2%) |  |  |  |
| Missing | 1 (1.7%) | 6 (5.6%) | 2 (2.4%) | 1 (5.3%) | 3 (2.9%) | 5 (13.5%) |  |  |  |
| **Intracranial pathology** |  |  |  |  |  |  | *χ2*(25) = 21.7067 | 0.6526 | 1 |
| none | 45 (75.0%) | 74 (69.2%) | 61 (74.4%) | 16 (84.2%) | 81 (78.6%) | 31 (83.8%) |  |  |  |
| OPG asymtomatic | 2 (3.3%) | 6 (5.6%) | 2 (2.4%) | 0 (0%) | 9 (8.7%) | 0 (0%) |  |  |  |
| OPG symptomatic | 4 (6.7%) | 10 (9.3%) | 3 (3.7%) | 1 (5.3%) | 3 (2.9%) | 1 (2.7%) |  |  |  |
| Glioma asymtomatic | 1 (1.7%) | 3 (2.8%) | 2 (2.4%) | 0 (0%) | 2 (1.9%) | 0 (0%) |  |  |  |
| Glioma symptomatic | 1 (1.7%) | 3 (2.8%) | 3 (3.7%) | 0 (0%) | 2 (1.9%) | 0 (0%) |  |  |  |
| other | 1 (1.7%) | 6 (5.6%) | 5 (6.1%) | 1 (5.3%) | 2 (1.9%) | 1 (2.7%) |  |  |  |
| Missing | 6 (10.0%) | 5 (4.7%) | 6 (7.3%) | 1 (5.3%) | 4 (3.9%) | 4 (10.8%) |  |  |  |
| **Psychoactive medication use** |  |  |  |  |  |  | *χ2*(10) = 13.2638 | 0.2093 | 1 |
| no | 56 (93.3%) | 93 (86.9%) | 71 (86.6%) | 14 (73.7%) | 82 (79.6%) | 30 (81.1%) |  |  |  |
| adhd | 3 (5.0%) | 4 (3.7%) | 7 (8.5%) | 3 (15.8%) | 14 (13.6%) | 4 (10.8%) |  |  |  |
| other | 1 (1.7%) | 4 (3.7%) | 3 (3.7%) | 2 (10.5%) | 5 (4.9%) | 3 (8.1%) |  |  |  |
| Missing | 0 (0%) | 6 (5.6%) | 1 (1.2%) | 0 (0%) | 2 (1.9%) | 0 (0%) |  |  |  |
| **SRS** |  |  |  |  |  |  |  |  |  |
| Age, median (Q1 - Q3)^a^ | 7.82 (6.00 - 9.86) | 7.00 (6.00 - 10.0) | 8.20 (5.76 - 11.7) | 7.13 (6.00 - 10.7) | 8.12 (5.87 - 11.1) | 8.30 (6.81 - 10.6) | *F*(5,326) = 0.6504 | 0.6614 | 1 |
| Missing | 10 (16.7%) | 18 (16.8%) | 15 (18.3%) | 5 (26.3%) | 23 (22.3%) | 5 (13.5%) |  |  |  |
| **ADOS** |  |  |  |  |  |  |  |  |  |
| Age, median (Q1 - Q3)^b^ | 7.10 (5.48 - 8.93) | 6.73 (5.21 - 8.58) | 6.67 (5.39 - 9.65) | 6.66 (3.93 - 10.3) | 6.79 (5.00 - 9.03) | 7.95 (6.73 - 11.3) | *F*(5,256) = 0.5059 | 0.7717 | 1 |
| Module |  |  |  |  |  |  | *χ2*(15) = 29.557 | 0.0136 | 0.109 |
| preverbal module | 0 (0%) | 0 (0%) | 0 (0%) | 0 (0%) | 0 (0%) | 0 (0%) |  |  |  |
| module 1 | 2 (3.3%) | 2 (1.9%) | 1 (1.2%) | 5 (26.3%) | 7 (6.8%) | 2 (5.4%) |  |  |  |
| module 2 | 8 (13.3%) | 17 (15.9%) | 14 (17.1%) | 2 (10.5%) | 19 (18.4%) | 2 (5.4%) |  |  |  |
| module 3 | 25 (41.7%) | 50 (46.7%) | 34 (41.5%) | 9 (47.4%) | 43 (41.7%) | 14 (37.8%) |  |  |  |
| module 4 | 0 (0%) | 1 (0.9%) | 1 (1.2%) | 0 (0%) | 1 (1.0%) | 2 (5.4%) |  |  |  |
| Missing | 25 (41.7%) | 37 (34.6%) | 32 (39.0%) | 3 (15.8%) | 33 (32.0%) | 17 (45.9%) |  |  |  |
| **CRS** |  |  |  |  |  |  |  |  |  |
| Age, median (Q1 - Q3)^b^ | 6.95 (5.19 - 8.71) | 7.12 (6.13 - 9.71) | 7.43 (6.09 - 11.3) | 6.64 (6.06 - 10.1) | 8.11 (5.65 - 9.81) | 8.30 (7.16 - 9.83) | *F*(5,205) = 1.2648 | 0.2805 | 1 |
| Missing | 26 (43.3%) | 53 (49.5%) | 41 (50.0%) | 11 (57.9%) | 47 (45.6%) | 19 (51.4%) |  |  |  |
| **CBCL** |  |  |  |  |  |  |  |  |  |
| Age, median (Q1 - Q3)^b^ | 6.49 (5.22 - 8.22) | 6.34 (5.00 - 8.08) | 6.67 (5.00 - 10.1) | 6.26 (5.47 - 10.4) | 6.56 (5.13 - 8.96) | 7.05 (5.50 - 10.0) | *F*(5,247) = 0.3792 | 0.8627 | 1 |
| Missing | 18 (30.0%) | 32 (29.9%) | 38 (46.3%) | 4 (21.1%) | 45 (43.7%) | 18 (48.6%) |  |  |  |

^a^ For analysis, this variable was square-root transformed. ^b^ For analysis, this variable underwent logarithmic transformation.

**Supplemental Table 2**: Cohort characteristics by a variant being truncating or not.

|  | ***NF1***x | ***NF1***p | **Comparisons** |  |  |
| --- | --- | --- | --- | --- | --- |
|  | ***(n* = 224)** | **(*n* = 68)** | **Statistic** | ***p*-value** | **Adjusted *p*-value** |
| **Sex** |  |  | *χ2*(1) = 0.0014 | 0.9697 | 1 |
| male | 118 (52.7%) | 36 (52.9%) |  |  |  |
| female | 106 (47.3%) | 32 (47.1%) |  |  |  |
| **Inheritance** |  |  | *χ2*(1) = 0.1865 | 0.6658 | 1 |
| de novo | 142 (63.4%) | 45 (66.2%) |  |  |  |
| familial | 72 (32.1%) | 20 (29.4%) |  |  |  |
| Missing | 10 (4.5%) | 3 (4.4%) |  |  |  |
| **Intracranial pathology** |  |  | *χ2*(5) = 2.3873 | 0.7934 | 1 |
| none | 166 (74.1%) | 49 (72.1%) |  |  |  |
| OPG asymtomatic | 15 (6.7%) | 2 (2.9%) |  |  |  |
| OPG symptomatic | 13 (5.8%) | 4 (5.9%) |  |  |  |
| Glioma asymtomatic | 5 (2.2%) | 1 (1.5%) |  |  |  |
| Glioma symptomatic | 5 (2.2%) | 1 (1.5%) |  |  |  |
| other | 10 (4.5%) | 1 (1.5%) |  |  |  |
| Missing | 10 (4.5%) | 10 (14.7%) |  |  |  |
| **Psychoactive medication use** |  |  | *χ2*(2) = 1.6092 | 0.4473 | 1 |
| no | 184 (82.1%) | 62 (91.2%) |  |  |  |
| adhd | 22 (9.8%) | 4 (5.9%) |  |  |  |
| other | 10 (4.5%) | 2 (2.9%) |  |  |  |
| Missing | 8 (3.6%) | 0 (0%) |  |  |  |
| **SRS** |  |  |  |  |  |
| Age, median (Q1 - Q3)^a^ | 8.00 (6.00 - 11.0) | 7.82 (6.00 - 10.2) | *F*(1,236) = 0.0287 | 0.8657 | 1 |
| Missing | 44 (19.6%) | 10 (14.7%) |  |  |  |
| **ADOS** |  |  |  |  |  |
| Age, median (Q1 - Q3)^b^ | 6.83 (5.00 - 9.06) | 7.10 (5.34 - 9.20) | *F*(1,184) = 0.3693 | 0.5442 | 1 |
| **ADOS Module** |  |  | *χ2*(3) = 0.5813 | 0.9007 | 1 |
| module 1 | 11 (4.9%) | 2 (2.9%) |  |  |  |
| module 2 | 36 (16.1%) | 10 (14.7%) |  |  |  |
| module 3 | 96 (42.9%) | 28 (41.2%) |  |  |  |
| module 4 | 2 (0.9%) | 1 (1.5%) |  |  |  |
| Missing | 79 (35.3%) | 27 (39.7%) |  |  |  |
| **CRS** |  |  |  |  |  |
| Age, median (Q1 - Q3)^b^ | 7.71 (5.91 - 9.85) | 6.80 (5.18 - 8.75) | *F*(1,145) = 1.714 | 0.1925 | 1 |
| Missing | 114 (50.9%) | 31 (45.6%) |  |  |  |
| **CBCL** |  |  |  |  |  |
| Age, median (Q1 - Q3)^b^ | 6.38 (5.00 - 8.96) | 6.66 (5.09 - 8.50) | *F*(1,191) = 0.009 | 0.9247 | 1 |
| Missing | 78 (34.8%) | 21 (30.9%) |  |  |  |

^a^ For analysis, this variable was square-root transformed. ^b^ For analysis, this variable underwent logarithmic transformation.

Abbreviations: *NF1*p, non-truncating; *NF1*x, truncating.

**Supplemental Table 3**: Cohort characteristics per variant location.

|  | **pre-GAP** | **intra-GAP** | **post-GAP** | **Comparisons** |  |  |
| --- | --- | --- | --- | --- | --- | --- |
|  | ***(n* = 27)** | **(*n* = 20)** | **(*n* = 21)** | **Statistic** | ***p*-value** | **Adjusted *p*-value** |
| **Sex** |  |  |  | *χ2*(2) = 5.7582 | 0.0562 | 0.4495 |
| male | 11 (40.7%) | 15 (75.0%) | 10 (47.6%) |  |  |  |
| female | 16 (59.3%) | 5 (25.0%) | 11 (52.4%) |  |  |  |
| **Inheritance** |  |  |  | *χ2*(2) = 7.9796 | 0.0185 | 0.1665 |
| de novo | 21 (77.8%) | 15 (75.0%) | 9 (42.9%) |  |  |  |
| familial | 5 (18.5%) | 4 (20.0%) | 11 (52.4%) |  |  |  |
| Missing | 1 (3.7%) | 1 (5.0%) | 1 (4.8%) |  |  |  |
| **Intracranial pathology** |  |  |  | *χ2*(10) = 9.3907 | 0.4955 | 1 |
| none | 17 (63.0%) | 17 (85.0%) | 15 (71.4%) |  |  |  |
| OPG asymtomatic | 0 (0%) | 1 (5.0%) | 1 (4.8%) |  |  |  |
| OPG symptomatic | 3 (11.1%) | 1 (5.0%) | 0 (0%) |  |  |  |
| Glioma asymtomatic | 1 (3.7%) | 0 (0%) | 0 (0%) |  |  |  |
| Glioma symptomatic | 1 (3.7%) | 0 (0%) | 0 (0%) |  |  |  |
| other | 0 (0%) | 1 (5.0%) | 0 (0%) |  |  |  |
| Missing | 5 (18.5%) | 0 (0%) | 5 (23.8%) |  |  |  |
| **Psychoactive medication use** |  |  |  | *χ2*(4) = 3.5713 | 0.4671 | 1 |
| no | 23 (85.2%) | 19 (95.0%) | 20 (95.2%) |  |  |  |
| adhd | 3 (11.1%) | 1 (5.0%) | 0 (0%) |  |  |  |
| other | 1 (3.7%) | 0 (0%) | 1 (4.8%) |  |  |  |
| **SRS** |  |  |  |  |  |  |
| Age, median (Q1-Q3)^a^ | 8.16 (6.00-13.4) | 7.37 (5.13-9.48) | 7.33 (6.05-9.71) | *F*(2,55) = 0.7705 | 0.4677 | 1 |
| Missing | 4 (14.8%) | 4 (20.0%) | 2 (9.5%) |  |  |  |
| **ADOS** |  |  |  |  |  |  |
| Age, median (Q1-Q3)^a^ | 7.74 (5.44-8.66) | 7.09 (5.34-9.20) | 6.97 (5.72-9.26) | *F*(2,38) = 0.0869 | 0.917 | 1 |
| **ADOS Module** |  |  |  | *χ2*(6) = 3.4145 | 0.7553 | 1 |
| module 1 | 1 (3.7%) | 1 (5.0%) | 0 (0%) |  |  |  |
| module 2 | 3 (11.1%) | 4 (20.0%) | 3 (14.3%) |  |  |  |
| module 3 | 10 (37.0%) | 8 (40.0%) | 10 (47.6%) |  |  |  |
| module 4 | 0 (0%) | 0 (0%) | 1 (4.8%) |  |  |  |
| Missing | 13 (48.1%) | 7 (35.0%) | 7 (33.3%) |  |  |  |
| **CRS** |  |  |  |  |  |  |
| Age, median (Q1-Q3)^a^ | 6.70 (4.93-8.19) | 6.33 (4.80-9.20) | 7.22 (5.98-8.81) | *F*(2,34) = 0.2159 | 0.8069 | 1 |
| Missing | 15 (55.6%) | 7 (35.0%) | 9 (42.9%) |  |  |  |
| **CBCL** |  |  |  |  |  |  |
| Age, median (Q1-Q3)^a^ | 6.89 (5.13-8.37) | 6.00 (4.86-7.19) | 6.80 (5.81-9.71) | *F*(2,44) = 1.0106 | 0.3723 | 1 |
| Missing | 7 (25.9%) | 8 (40.0%) | 6 (28.6%) |  |  |  |

^a^ For analysis, this variable underwent logarithmic transformation.
